# Cytotoxic and Apoptotic Potential of *Rheum turkestanicum* Janisch Root Extract on Human Cancer and Normal Cells 

**Published:** 2013

**Authors:** Farideh Shiezadeh, Seyed Hadi Mousavi, Mohammad Sadegh Amiri, Mehrdad Iranshahi, Zahra Tayarani-Najaran, Gholamreza Karimi

**Affiliations:** a *Pharmacological Research Centre of Medicinal Plants, School of Medicine, Mashhad, University of Medical Sciences, Mashhad, Iran. *; b *Department of Biology, Payame Noor University, 193953697- Tehran, Iran. *; c *Biotechnology Research Center and School of Pharmacy, Mashhad University of Medical Sciences, Mashhad, Iran.*; d *Department of Pharmacodynamics and Toxicology, School of Pharmacy, Mashhad, University of Medical Sciences, Mashhad, Iran.*; e *Medical Toxicology Research Center and Pharmacy School, Mashhad University of Medical Sciences, Mashhad, Iran. *

**Keywords:** *Rheum turkestanicum *Janischew., Polygonaceae, Cytotoxicity, Apoptosis, Cancer

## Abstract

*Rheum turkestanicum *Janischew. (Polygonaceae) is a plant that grows in central Asia and in north-east of Iran. Traditionally, people use roots of *R. turkestanicum *as an anti-diabetic and anti-hypertensive as well as anticancer agent. In this study the cytotoxicity and apoptogenic properties of ethyl acetate (EtOAc), *n-*hexane and H_2_O extracts from *Rheum turkestanicum *Janischew. (Polygonaceae) root were determined against HeLa and MCF-7 cell lines and human blood lymphocytes.

Malignant and non-malignant cells were cultured in RPMI 1640 medium and incubated with different concentrations of plant extracts. Cell viability was measured by MTS assay. Apoptotic cells were evaluated using PI staining of DNA fragmentation by flow cytometry (sub-G1 peak). The degree of DNA fragmentation was analyzed using agarose gel electrophoresis based on the formation of inter-nucleosomal units. The expression of apoptosis-related protein Bax and PARP cleavage were detected by Western blotting.

EtOAc and *n-*hexane extracts decreased cell viability in malignant but not in non-malignant cells, as a concentration and time dependent manner. EtOAc extract induced a sub-G1 peak in flow cytometry histogram of treated cells compared to the control. DNA fragmentation indicating apoptotic cell death was involved in *R. turkestanicum *induced toxicity and cleaved PARP fragment was also detected.

In conclusion, this is the first report on the cytotoxic effects of *R. turkestanicum *in which apoptosis played an important role. However, further evaluations are needed to fully understand the possible anti-tumor properties.

## Introduction

Most cancer chemotherapy agents currently in clinical use have originated from plants or are analogs of plant-derived compounds (1-2). Only 41.4% of the compounds reported in natural product databases, such as Napralert, have been studied from a biological perspective (3). The use of naturally occurring compounds with chemopreventive properties attracted much interest in chemotherapy and treatment of cancers. The anti-cancer properties of a multitude of medicinal herbs mediated through different mechanisms include altered carcinogen metabolism, induction of DNA repair systems, immune activation and suppression of cell cycle progression/induction of apoptosis. While cancer cell death/apoptosis could be considered a convergence point of all anti-neoplastic therapies, direct pro-apoptotic effects have been reported for bioactive phytochemicals (4).

*Rheum *species (Polygonaceae) have a long history as medicinal plants in traditional Chinese medicine. *Rhei Rhizoma *(Dahuang) is a Chinese herbal medication that has traditionally been prescribed for its purported purgative and anti-inflammatory properties. According to the Chinese Pharmacopoeia, *Rhei Rhizoma *is derived from one of three *Rheum *species, *R. palmatum*, *R. tanguticum*, and *R. officinale*. The main active ingredients of the *Rheum *species are a series of anthraquinones, dianthrones, glycosides and tannins. The anthraquinone derivatives include emodin, rhein, chrysophanol, physcion, alizarin, citreorosein, and aloe-emodin (5). 

Recently, the antitumor activity of these plants has attracted much attention. Roots of *R. emodi *are reported to have antibacterial, antifungal, laxative, diuretic, and *in-vivo *inhibitory effects towards P388 leukemia in mice. Aqueous and methanolic extracts of *R. emodi *showed concentration-dependent cytotoxicity when tested in MDA-MB-435S (human breast carcinoma) and Hep3B (liver carcinoma) cell lines (6). 

*R. officinale *Baill. is one of the herbs commonly used in formulas of Traditional Chinese Medicine (TCM) prescribed to cancer patients. It has been reported to have anti-tumor activity with hepatocarcinoma and to significantly inhibit the proliferation of A549 and MCF-7 cells in vitro, confirmed by the cell viability and colony formation assays. Its cytotoxic activity is likely to be due to the induction of apoptosis (7). The *n-*hexane extract of *R. undulatum *L. has been shown to have anti-cancer properties in several cancers *in-vivo *and *in-vitro *(8). Numerous reports have shown that emodin, aloe-emodin and rhein have anti-proliferative effects on many kinds of cancer cell lines such as HER-2/neu over expressing breast cancer, leukemia, hepatoma, human myeloma and lung cancer with the involvement of apoptosis or programmed cell death (9-12).

*Rheum turkestanicum *(Polygonaceae) is a plant that grows widely in central Asia and also in north-east of Iran. Traditionally, people use roots of *R. turkestanicum *as an anti-diabetic and anti-hypertensive as well as anticancer agent. In spite of the traditional use of *R. turkestanicum *as an anti-cancer plant and widespread researches about the cytotoxic and antitumor effects of some species of the *Rheum *genus, there is not any reported literature on *R. turkestanicum*. 

The aim of this study was to investigate the *in-vitro *cytotoxic activity of ethyl acetate (EtOAc), *n-*hexane and H_2_O extracts from *Rheum turkestanicum*. Therefore, in an attempt is sought to study cytotoxic properties of three different extracts (ethyl acetate (EtOAc), *n-*hexane and H2O) of *R. turkestanicum *root on two cancer cell lines, including human cervix carcinoma cell line (HeLa) and human breast cancer cell line (MCF-7) and non-malignant cells (Human Blood Lymphocytes). The role of apoptosis in *R. turkestanicum *induced cytotoxicity on cancer cell lines has also been reported. In this study, we used Paclitaxel as a positive control (13).

## Experimental

*Reagents*

MTS (3-(4, 5-Dimethylthiazol-2-yl) -5-(3-carboxymethoxyphenyl) -2- (4-sulfophenyl) -2H-tetrazolium) from Promega (Madison, WI, USA); (RPMI-1640) and FCS from Gibco; Lympholyte®-H from Cedarlane (Canada); *β*-actin and PARP antibodies, anti- rabbit IgG and HRP linked antibody from Cell Signaling technology (Boston, USA); ECL Western blotting detection reagent from Bio-RaD (USA); the fluorescent probe propidium iodide (PI), protease inhibitor cocktail, phosphatase inhibitor cocktail, sodium citrate, Triton X-100, phenylmethylsulfonyl fluoride and QuantiPro BCA assay kit were purchased from Sigma (Steinheim, Germany). 

*Plant materials*

**Figure 1:**
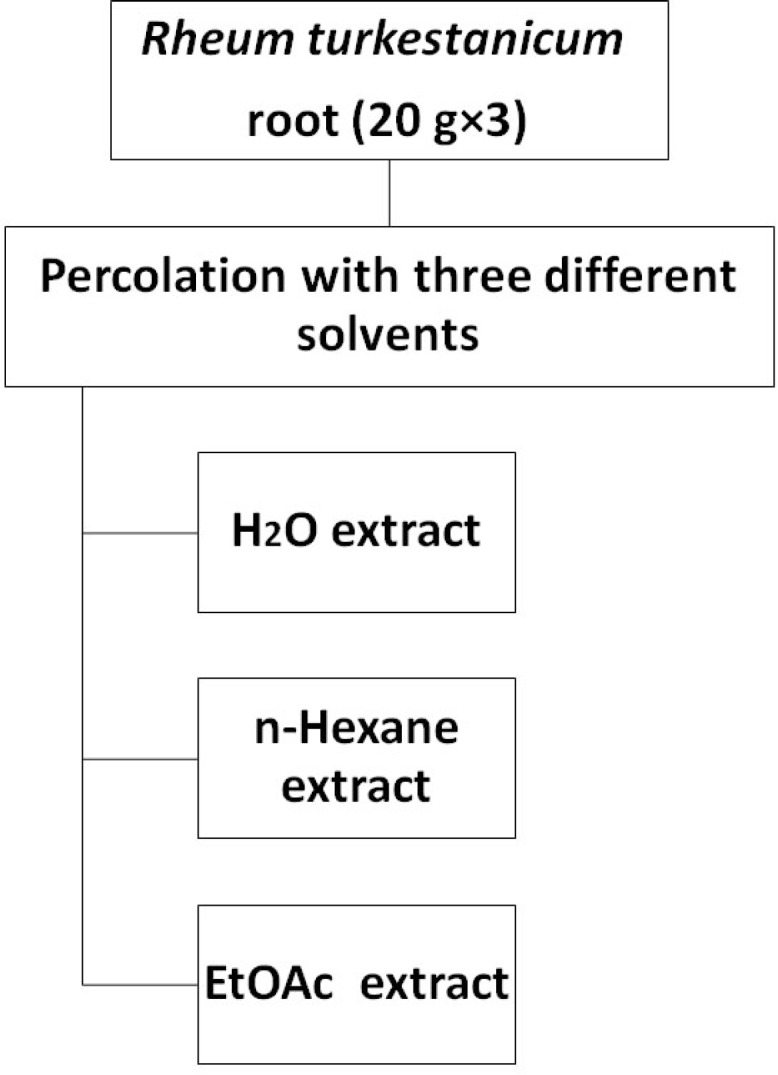
Extraction scheme of *R. turkestanicum*

The root of *R. turkestanicum *was collected from Chenar, a village in Zavin Rural District, Kalat County, Razavi Khorasan Province, Iran.The plant was identified by M. R. Joharchi, from Ferdowsi University of Mashhad Herbarium. Voucher specimen (No. 42082) was deposited in Ferdowsi University of Mashhad Herbarium. Dried *R. turkestanicum *root (20 g×3) was ground into fine powder and then was percolated with 100 ml of each solvent (EtOAc, *n-*hexane and H_2_O). After 24 h, the solutions were centrifuged at 2700 g for 3 min. The supernatants were collected and the residues were re-extracted for one more time with the same volume of solvents (Figure 1). The whole extract were filtered and the solvents were evaporated under reduced pressure at 40–45 °C, each extract was then stored at -20 °C over night, and then it was concentrated by freeze drier. Again the extract kept at -20 °C. The dried extrcts were dissolved in dimethylsulfoxide (DMSO) and were then screened for tumor cell growth inhibition. 

*Cell culture*

HeLa and MCF-7 cells were obtained from the Pasteur Institute (Tehran, Iran) and lymphocytes isolated from human peripheral blood using the Lympholyte®-H (a density gradient separation medium) according to manufacturer’s protocol and maintained at 37 °C in a humidified atmosphere (90%) containing 5% CO_2_. Cells were cultured in Roswell Park Memorial Institute-1640 (RPMI-1640) with 10% (v/v) fetal bovine serum, 100 U/mL penicillin, and 100 μg/mL streptomycin. For each concentration and time course study, there was a control sample which remained untreated and received the equal volume of medium.

*Leukocyte culture*

Human umbilical cord blood samples (50 mL) were collected from a fresh umbilical cord attached to the placenta by gravity flow in a sterile 50 mL syringe containing citrate buffer as an anticoagulant. The sample was diluted with an equal volume of phosphate buffered saline (PBS) and then layered over Lympholyte®-H a density gradient separation solution, and centrifuged at 800 g for 20 min at room temperature. The mononuclear cell layer was removed, washed twice in PBS and resuspended in RPMI 1640 medium supplemented with 2 mM glutamine (Sigma Chemical Co.), antibiotics and 10% FCS. Leukocytes (5×10^4^ cells per well) were incubated with 

various concentrations of *R. turkestanicum *in 96-well plates for 48 h. This study protocol was approved by the ethical committee of Mashhad University of Medical Sciences.

*Cell viability*

The MTS [3- (4,5-Dimethylthiazol-2-yl)-5-(3-carboxymethoxyphenyl)-2- (4-sulfophenyl) -2H-tetrazolium] growth inhibition assay (14) was performed according to the instructions provided by the manufacturer (Promega). Briefly, the cells were seeded (10^4^ cell/well) onto flat-bottomed 96-well culture plates. The cells were either treated with ethyl acetate, *n-*hexane and H_2_O extracts (15-500 μg/mL) over different incubation periods (24, 48 and 72 h), or remained as untreated controls. At the end of each time point, fresh complete medium containing 10 μL of MTS solution was added and further incubated for 2 h. Optical density of each culture was then recorded at 490 nm using an ELISA reader. Each experiment was performed in triplicate and all the extracts were compared with Paclitaxel (0.7 μM) as a positive control. Results are expressed as the percentage growth inhibition with respect to the untreated cells.

*Apoptosis*

*PI staining*

Apoptotic cells were detected using PI staining of treated cells followed by flow cytometry to detect the so-called sub-G1 peak (15). Briefly, malignant cells were cultured overnight in a 24-well plate and treated with extracts (EtOAc and *n-*hexane) for 48 h. Floating and adherent cells were then harvested and incubated at 4 °C overnight in the dark with 750 μL of a hypotonic buffer (50 μg/mL PI in 0.1% sodium citrate plus 0.1% Triton X-100) before flow cytometric analysis using a FACScan flow cytometer (Becton Dickinson). About 10,000 events were acquired with FACS. 

*Western blotting analysis*

HeLa cells were treated with 150 and 200 μg/mL of the EtOAc and *n-*hexane extracts of *R. turkestanicum *for 48 h. The cells were harvested and rinsed with ice-cold PBS. The cell pellet was resuspended in a lysis buffer containing 50 mM Tris-HCl (pH 7.4), 150 mM NaCl, 1% triton-X100, 1 mM EDTA, 0.2% SDS, 1% protease inhibitor cocktail, 1% phosphatase inhibitor cocktail and 1 mM phenylmethylsulfonyl fluoride and left on ice for 30 min. After centrifugation at 10000 rpm for 20 min at 4 °C, the cell lysate was collected and protein concentration was determined according to the BCA detection kit (16). Equal amounts of proteins were subjected to 12.5% SDS–PAGE (w/v). The proteins were transferred to a polyvinylidene fluoride (PVDF) membrane and subjected to immunoblotting using *β*-actin antibody and PARP antibody as primary antibodies and anti- rabbit IgG, HRP linked antibody, as secondary antibodies. PARP cleavage in HeLa cells were detected by enhanced chemi luminescence using the ECL western blotting detection reagent. 

*DNA fragmentation analysis*

To evaluate oligonucleosomal fragmentation, genomic DNA was extracted as previously described (17). MCF-7 cells were treated with different concentrations of each EtOAc extract for 48 h in RPMI 1640 supplemented with 10% (v/v) fetal calf serum, 100 U/mL penicillin and 100 mg/ mLstreptomycin. 

The formation of high molecular weight and oligonucleosomal DNA fragments was examined by agarose gel electrophoresis. Cells (10^6^ cells) were seeded onto 6-well plates and treated for 48 h. The cells were collected by centrifugation at 1100 rpm for 7 min. The DNA from treated and untreated cells was extracted as explained below: cells were incubated with 50 μL of lysis buffer (20 mM Tris, 20 mM EDTA, 200 mM NaCl and 1% SDS) and 2 μL RNase A (500 μg/mL) for 15 min at 37 ºC. The cells were further incubated at 37 ºC for 15 min after adding 2.5 μL of 10 mg/mL Proteinase K, which had been preheated at 37 ºC for 30 min. The lysate was mixed with 10 mL of loading solution (30% ficoll, and 1% bromophenol blue in TBE), and then the DNA samples were separated in 2% agarose gel electrophoresis at 50 V, 3 h and visualized with ethidium bromide using Gel Documentation System (Far Gene Pouyesh, Tehran, Iran). 

*Statistical analysis*

One way analysis of variance (ANOVA) and Bonferroni’s post hoc were used for data analysis. All results were expressed as mean±SEM and p-values below 0.05 were considered statistically significant. 

## Results

*Cytotoxicity of various extracts of R. turkestanicum*

**Figure 2:**
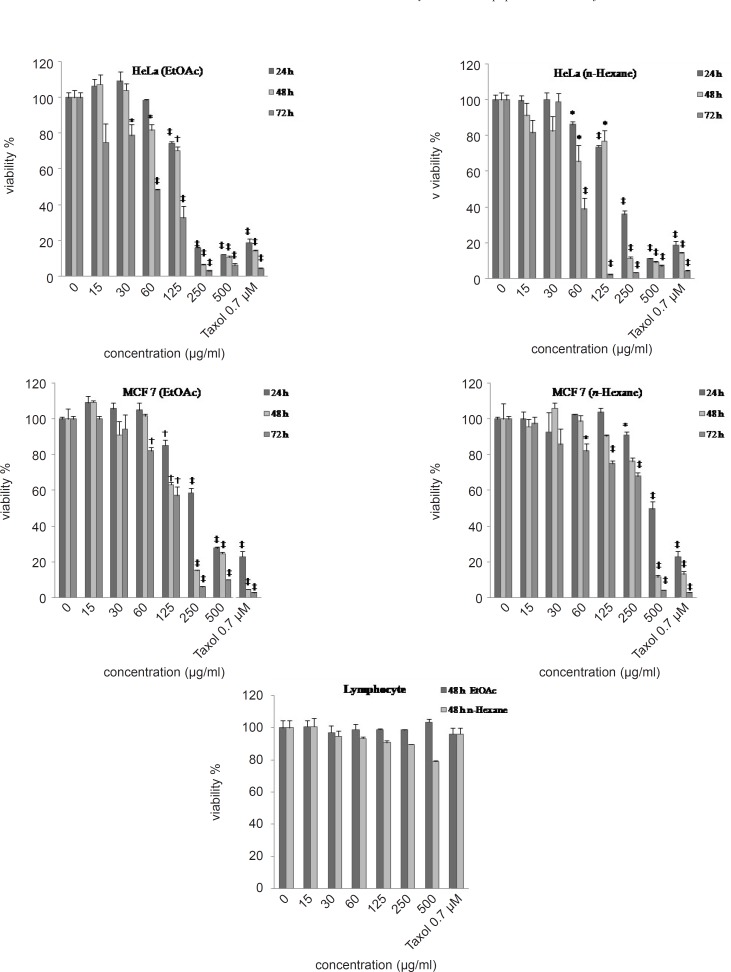
Dose-dependent growth inhibition of malignant (MCF-7 and HeLa cells respectively) and non-malignant control cells (human blood lymphocytes) by the EtOAc, *n-*hexane and H_2_O extracts (15–500 μg/mL) after 24, 48 and 72 h. Viability was quantitated by MTS assay. Results are mean ± (n = 3). *p < 0.05, †p < 0.01 and ‡p < 0.001 compared to control

Malignant cells and human blood lymphocytes (as non-malignant control cells) were incubated with various concentrations of EtOAc, *n-*hexane and H2O extracts of *R. turkestanicum *(15- 500 μg/mL) for 24, 48 and 72 h. EtOAc and *n-*hexane extracts decreased cell viability in malignant cells but not in non-malignant cells, as a concentration and time-dependent manner (Figure 2). 

This toxicity was consistent with morphologic changes including reduction in cell volume and rounding (data was not shown). Doses inducing 50% cell growth inhibition (IC_50_) against HeLa and MCF-7 cells are presented in Table 1. 

**Table 1:** Doses of toxic extracts of *R. turkestanicum *inducing 50% cell growth inhibition (IC_50_) against malignant cell lines. Cells incubated with different concentration of extractsfor 48 h. IC_50_ values were expressed as the mean ± SEM (n = 3).

**Cell line/ Extract **	***n-*** **Hexane **	**EtOAc **
MCF-7	320	155
HeLa	130	150

*Role of apoptosis*

*PI staining*

**Figure 3:**
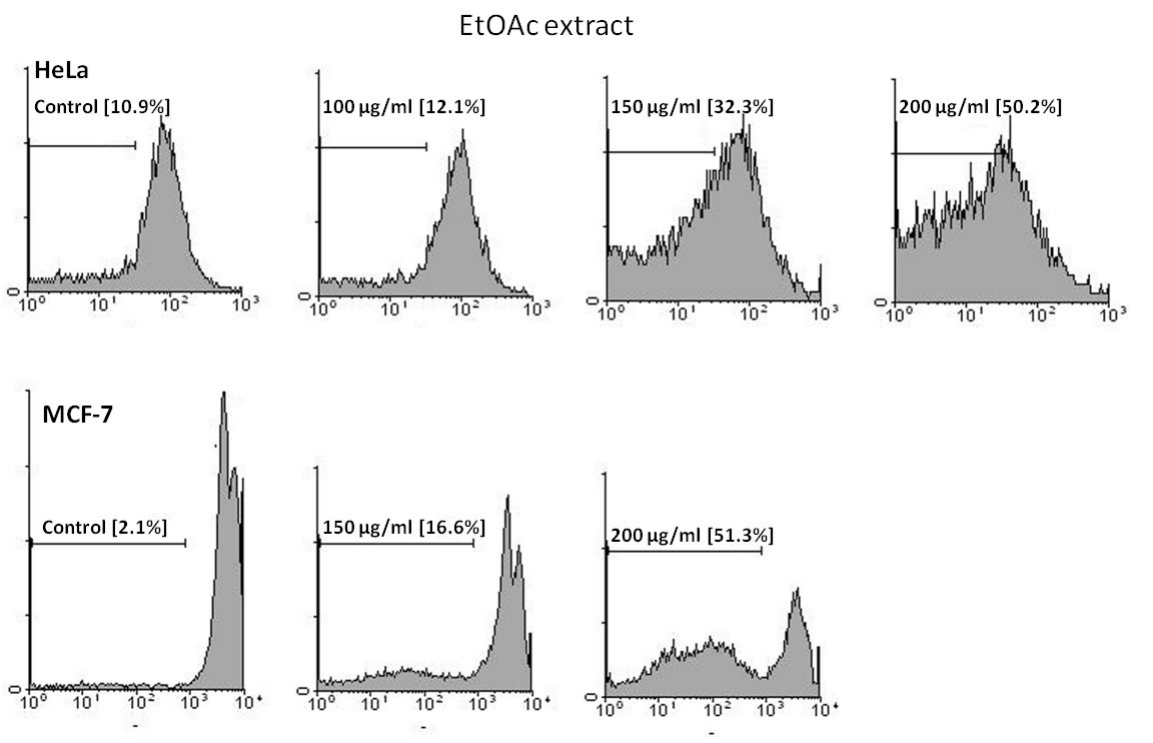
Flow cytometry histograms of apoptosis assays by PI method in HeLa and MCF-7 cells. Cells were treated with 100, 150 and 200 μg/mL in HeLa and 150 and 200 μg/mL in MCF-7 cells by EtOAc extract for 48 h. Sub-G1 peak as an indicative of apoptotic cells, was induced in EtOAc extract-treated but not in control cells indicating involvement of an apoptosis in EtOAc extract -induced cell death

The proportion of apoptotic cells was measured with PI staining of DNA fragmentation by flow cytometry. EtOAc extract induced a sub- G1peak (one of the reliable biochemical markers of apoptosis) in flow cytometry histogram of treated cells compared to control (Figure 3). 

*Cleavage of DNA and PARP*

**Figure 4:**
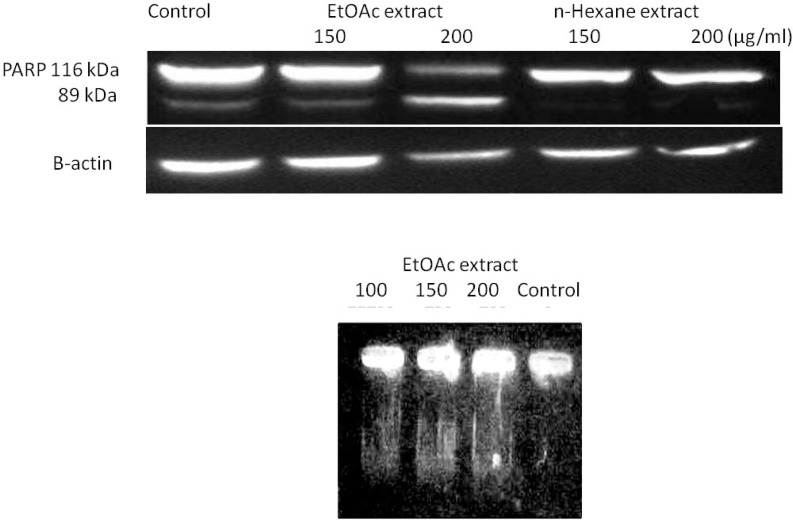
(A) Confirmation of apoptosis mediated cell death in MCF-7 cells through observation of DNA laddering using DNA fragmentation assay on treated cells with EtOAc for 48 h followed by analysis of extracted DNA on 2% (w/v) agarose gel electrophoresis. No distinct fragmentations were observed in control lane indicating the absence of apoptosis as opposed to lanes 2, 3 and 4 (B) PARP degradation was observed in lane 200 μg/mL EtOAc. Equal amounts of cellular proteins were subjected to SDS-PAGE, and PARP degradation from its native form (116 kDa) to the cleaved form (89 kDa) was detected by Western blot analysis

We also examined the induction of apoptosis in MCF-7 and HeLa cells through the occurrence of DNA fragmentation and cleavage of PARP proteins respectively. We observed that DNA extracted from untreated MCF-7 cells showed no fragmentation, while DNA from ethyl acetate-treated cells (48 h) showed DNA laddering as a result of endonuclease action at sites between nucleosomes (Figure 4). The suspected involvement of caspase-3 activation in EtOAc induced apoptosis was also validated in HeLa cells through the expression and degradation of a nuclear protein, PARP. 

Proteolytic cleavage of full length PARP from a 116 kDa polypeptide to a 24 kDa and 89 kDa segment is a typical marker for the onset of apoptosis (Figure 4). Therefore, the apoptosis inducing effects of EtOAc on malignant cells were confirmed. 

## Discussion

We tested ethyl acetate, *n-*hexane and H_2_O extracts from *R. turkestanicum *for its ability to inhibit the growth of HeLa and MCF-7 cells. We also tested the extracts for the ability to induce apoptosis in the mentioned cell lines. Reduced metabolic activity due to the *R. turkestanicum *extract was demonstrated using MTS. Apoptosis induction was demonstrated by sub-G1 peak in flow cytometry histogram of treated cells compared to control. DNA fragmentation indicating apoptotic cell death was involved in *R. turkestanicum*-induced toxicity and cleaved PARP fragment was also detected. 

In ancient times plants were used to treat various kinds of diseases (10). In traditional cultures herbal plants and plant-derived medicines have been commonly used and today they have become increasingly popular as natural alternatives (18). Nowadays, the origins of 60% of anticancer drugs are from natural products and plant derivatives such as antibiotics. As a result natural products have gained much attention as vital sources for developing of valuble effective anticancer agents (19). There are some reports of the cytotoxic activity of the *Rheum *species. Although there have been numerous studies on the effects of different *Rheum *species extracts and isolated phytochemicals on cancer cells, none have studied the effects of *R. turkestanicum. *

According to our results, the EtOAc and *n-*hexane extracts of *R. turkestanicum *caused a concentration and time-dependent inhibition of growth of both malignant cell lines with high IC_50_ values. The same dose of extract had little, if any, effect on lymphocytes. In fact, 15-500 μg/ mL of *R. turkestanicum *extract did not induce the same inhibitory effects in lymphocytes that were observed in tested tumor cell lines. The normal cells also failed to undergo the typical morphological changes seen following treatment of the HeLa and MCF-7 cells. This suggests that the *R. turkestanicum *extract may not affect normal cells, thus warranting *in-vivo *studies. 

Using PI labeling, DNA fragmentation and Western blot analysis for PARP cleavage, we demonstrate that apoptosis is involved in *R. turkestanicum*-induced cell death. *R. turkestanicum *extracts exerted their growth arrest on treated cells by increasing the cells at subG1-phase and DNA laddering implying that *R. turkestanicum *extracts causes separating PARP into a 24 kDa and 89 kDa segment and subsequently initiating DNA fragmentation. 

Apoptosis is a critical pathway occurs in development, immunological competence, and homeostasis. Cellular hallmark of apoptosis includes marked changes in morphology, including chromatin condensation, membrane blebbing, nuclear breakdown, and the appearance of membrane-associated apoptotic bodies, internucleosomal DNA fragmentation, as well as cleavage of poly (ADP-ribose) polymerase (PARP) (20-21). PARP catalyzes the poly (ADP-ribosyl) action of a variety of nuclear proteins with NAD (Nicotinamide adenine dinucleotide) as substrate. PARP cleavage into 89- and 24-kDa segments that contain the active site and the DNA-binding domain of the enzyme basically inactivates the enzyme by destroying its ability to respond to DNA strand breaks. Because it is activated by binding to DNA ends or strand breaks, PARP was suggested to contribute to drug-induced apoptosis in a variety of cells by depleting the cell of NAD and ATP after cleavage (22-24). 

The activation of caspase-3 in treated cancer cells during apoptosis results PARP cleavage, and activation of caspase activated DNase (CAD), subsequently causing DNA fragmentation, which is one of characteristics of apoptosis (25, 26). Thus, the presence of DNA ladder on agarose gel is due to elevation of caspases after *R. turkestanicum *treatments. 

This is the first report on the cytotoxic effects of *R. turkestanicum *in which apoptosis played an important role. It is also worth mentioning that according to the MTS results, Paclitaxel (0.7 μM) leaded to a more intense decrease in cell viability on human cancer cell line. Relatively high IC_50_ values may be a function of cell type used and short duration of exposure to extracts. Furthermore, anti-proliferative effects may be intensified in an *in-vivo *model. Further in vitro assays on some other malignant cell lines, phytochemical studies on pure compounds isolated from *R. turkestanicum *and *in-vivo *evaluations are suggested to confirm the result obtained in this study.
